# A New Method of Whitening Bones

**Published:** 1851-12

**Authors:** Ellerslie Wallace

**Affiliations:** Demonstrator of Anatomy in Jefferson Medical College, Philadelphia; 92 S. 11th St.


*A New Method of Whitening Bones.* By ELLERSLIE WALLACE, M. D., Demonstrator of Anatomy in Jefferson Medical College, Philadelphia.

To the Editors of the Medical Examiner.

*Gentlemen,*—During the past year, I have used sulphuric ether for the purpose of extracting the greasy matters from bones of which I have desired to make preparations, and have uniformly found it entirely satisfactory. I have used it for entire skeletons where they have been of value.

Twenty-five or thirty pounds of ether (which can be obtained for 18 cts. per lb.,) is enough for a skeleton, if the bones be closely packed in a proper case. After pouring the ether on them until they are entirely covered, they may be left for some hours, or a day; then removing them, they should be allowed to dry thoroughly. This process should be repeated as often as may be necessary.

Six immersions have been enough for a very greasy skeleton. It is prudent to wash the ether before using it, to remove free acid, and we may have the ether re-distilled after it is saturated with the oil. To morbid specimens, as of caries, &c., it is admirably adapted, as it removes the grease entirely, without injuring the delicate structure at all, which is not the case, as we all know, with any of the ordinary alkaline solutions.

Very truly, yours, ELLERSLIE WALLACE.

92 *S.* 11*th St.*, *Nov.* 1*st*, 1851.

